# Effectiveness of antibiotic prophylaxis in polytrauma patients: a systematic review and meta-analysis

**DOI:** 10.1007/s00068-025-02789-8

**Published:** 2025-02-13

**Authors:** Karolina Dahms, Kelly Ansems, Julia Dormann, Eva Steinfeld, Heidrun Janka, Maria-Inti Metzendorf, Thomas Breuer, Carina Benstoem

**Affiliations:** 1 Department of Intensive Care Medicine and Intermediate Care, Medical Faculty, RWTH Aachen University, Aachen, Germany; 2 Institute of General Practice, Medical Faculty of the Heinrich-Heine- University Dusseldorf, Dusseldorf, Germany; 3 Department of Intensive Care Medicine and Intermediate Care Medical Faculty, RWTH Aachen University, Pauwelsstr. 30, D-52074 Aachen, Germany

**Keywords:** Antibiotic prophylaxis, Intensive care unit, ICU, Polytrauma, Multiple trauma

## Abstract

**Purpose:**

The use of antibiotic prophylaxis in trauma patients, particularly to prevent ventilator-associated pneumonia (VAP), is debated due to rising antibiotic resistance. Therefore, this systematic review evaluated the safety and effectiveness of antibiotic prophylaxis compared to placebo or standard care on clinical outcomes in adult polytrauma patients.

**Methods:**

We searched PubMed and the Cochrane Central Register of Controlled Trials to identify completed and ongoing studies from database inception to April 20, 2023. Eligible studies included systematic reviews and randomized controlled trials (RCTs) comparing antibiotic prophylaxis to placebo or standard care in adult polytrauma patients admitted to the intensive care unit (ICU).

**Results:**

Of 1237 identified records, three RCTs involving 256 patients (n_antibiotics_ = 176, n_control_ = 165, mean age 37.4 years, 81.6% male) were included. Antibiotic prophylaxis showed little or no effect on all-cause mortality compared to placebo or standard care (RR 1.01, 95% CI 0.55–1.85; RD 2 more per 1000, 95% CI -79 to 150; 2 studies, 209 participants; *I*^2^ = 0%; very low certainty of evidence).

**Conclusion:**

The results indicate that antibiotic prophylaxis has no significant effect on mortality and clinical status compared with placebo or standard care in adult polytrauma patients but may reduce the risk of VAP. However, the evidence is outdated and of very low certainty, with insufficient data to draw definitive conclusions regarding efficacy. Therefore, high-quality, up-to-date research is urgently needed to support clinical decision-making, and current interpretations should be treated with caution.

## Introduction

Polytrauma, a condition characterized by severe multiple injuries, has long been a subject of concern in the medical community due to its association with a high mortality rate [1]. After 48 h of hospital stay, the mortality is particularly affected by systemic inflammatory response syndromes (SIRS), sepsis, and single and multiple organ failure (MOF) [2]. Among the many challenges faced by polytrauma patients, an increased risk of hemorrhage [3] and infections, such as ventilator associated pneumonia (VAP), is a significant threat to their recovery, as it is associated with an increased hospital stay, additional surgeries, and increased morbidity and mortality [4, 5, 6].

In this context, antibiotic prophylaxis is considered a possible solution [7, 8]. Selective decontamination of the digestive tract (SDD) is a prophylactic approach for patients in the intensive care unit (ICU). It has shown promise in reducing infection rates, especially in patients who require mechanical ventilation for over 48 h and often face the risk of developing VAP as a result of bacteria acquired during their hospital stay [9, 10, 11]. VAP and other acquired infections not only contribute to higher mortality rates but also pose significant challenges in treatment [8, 12].

However, the use of antibiotic prophylaxis remains a contentious topic in the medical field, especially in view of the alarming rise in antibiotic resistance. Antibiotic resistance is notably elevated within ICUs with outbreaks involving multidrug-resistant organisms [13, 14]. These challenges raise questions regarding the long-term effectiveness of antibiotic prophylaxis.

To provide current evidence on the efficacy of antibiotic prophylaxis, the objective of this systematic review is to assess the safety and effects of antibiotic prophylaxis compared to placebo or standard care on clinical outcomes in adult polytrauma patients in the ICU.

## Methods

This review is part of a guideline project that aimed to summarize the current evidence in the field of polytrauma to formulate specific recommendations. All studies that were conducted as part of this project used the same methodology, which was consented within the guideline group.

### Eligibility criteria

We included studies comparing antibiotic prophylaxis to standard care or placebo in adult polytrauma patients admitted to the ICU who met the following inclusion criteria:

- Age of the included patients is ≥ 18 years.
- Polytrauma is defined as a simultaneous injury to multiple body regions or organ systems, at least one or more of which, in combination, is life-threatening.
- Randomized controlled trial (RCT) or systematic review that includes RCTs.
- Language of publication: English or German.
- No multiple publication without additional information.
- Publication accessible as full text.
- Comparison of use of validated handover tools with no use.

### Search strategy

We conducted a systematic search developed by two experienced information specialists in PubMed and the Cochrane Central Register of Controlled Trials (CENTRAL) from inception of each database to April 24, 2023. The details of the search strategy are provided in the Appendix No 1. In addition, we searched reference lists of the included studies to identify other potentially eligible studies.

### Study selection

We imported the records from the systematic search into the Rayyan Systematic Review App (Rayyan, Cambridge, MA, USA). Three authors independently screened the titles and abstracts of the potential studies. Included full-text study publications were retrieved, imported into Microsoft Excel (Microsoft, Redmond, WA, USA), and screened by two authors independently. The reasons for exclusion of ineligible studies were recorded. Any disagreements were resolved through discussion or, if required, consultation with a third author if required.

### Data collection process

One reviewer extracted study and outcome data into a customized data collection form developed in Microsoft Excel, which was checked by a second investigator. Any disagreements were resolved by discussion or by consulting a third review author if necessary.

The following data were obtained:

1. Study characteristics: authors, publication date, and study design.
2. Participants characteristics: number of included participants, gender, age.
3. Clinical outcomes: all-cause mortality (day 28, day 60, time to-event, and up to longest follow-up), clinical status (duration of mechanical ventilation, need for mechanical ventilation), length of stay, serious adverse events (SAE), adverse events (AE), infections, quality of life.

We transmitted the outcome data into a statistical software (RevMan 5.3, Cochrane, London, England). Missing data resulted in the exclusion of the study from the analysis of the missing outcomes.

### Study risk of bias assessment

The risk of bias of the included studies was independently assessed by two authors using the Risk of Bias 2 (RoB 2) tool (Cochrane, London, England). This tool addresses five domains of bias (randomization process, deviations from intended interventions, missing outcome data, measurement of the outcome, and selection of the reported results). The signaling questions of the tool were used to make a judgement according to the available options. We used the algorithms proposed in RoB 2 to assign each domain and the overall risk of bias to a level of bias: low risk of bias, some concerns, and a high risk of bias. Any disagreements between the reviewers were resolved by discussion or by involvement of another author.

### Synthesis methods

Descriptive statistics were used to summarize demographics. Meta-analyses were performed only if the clinical and methodological characteristics of individual studies were sufficiently homogeneous. Data entry into the RevMan software was checked for accuracy by a second review author. Outcome data were pooled using the random-effects model, as we anticipated that the true effects would be related, but not the same, for the studies included in our review. For dichotomous data, we performed analyses using the Mantel–Haenszel method under a random-effects model to report pooled risk ratios (RR) with 95% confidence intervals (CI). For continuous outcomes, we calculated mean differences with 95% CIs. Forest plots were used to summarize the effects of the individual studies. When data was lacking or incomplete for analysis, such information was reported narratively. A p-value of < 0.05 was considered as statistically significant. The data were analyzed using the Cochrane methodology.

We intended to explore potential publication bias by generating a funnel plot and statistically testing this by conducting a linear regression test for meta-analyses involving at least 10 trials, as recommended in the Cochrane Handbook for Systematic Reviews of Interventions [15]. We considered *P* < 0.1 as significant for this test.

### Certainty assessment

We used the GRADEpro Guideline Development Tool Software (McMaster University and Evidence Prime Inc., Hamilton, Ontario, Canada) to create a summary of findings table and evaluated the certainty of the evidence using the GRADE approach for interventions evaluated in RCTs.

## Results

### Study selection

**Fig. 1:**
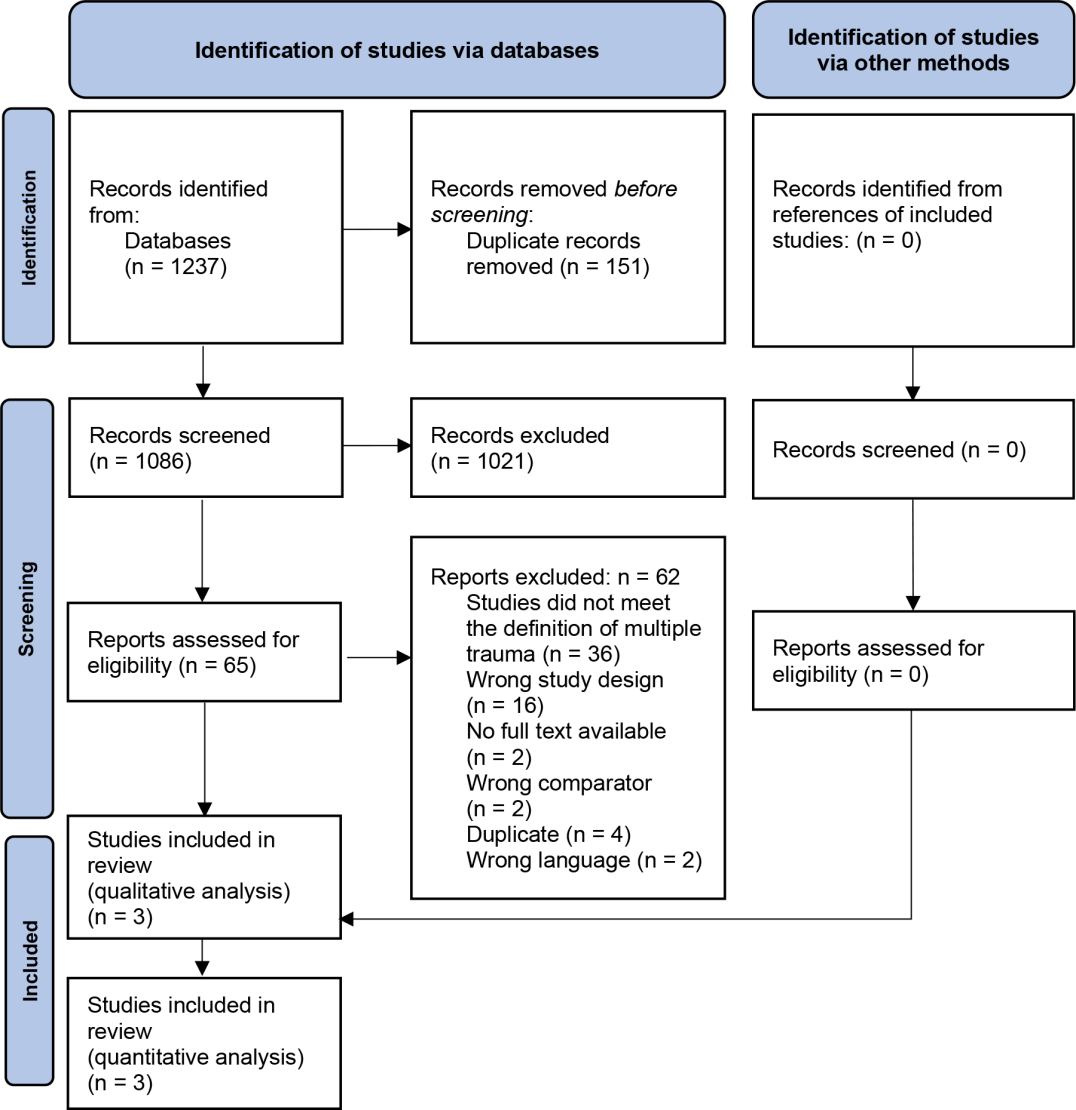
Flowchart of the systematic review selection process

The systematic search identified 1,145 records. After removing duplicates, we screened 1,086 records based on title and abstract; 1,021 studies did not meet the pre-specified inclusion criteria and were therefore excluded. We screened the full texts, trial register entries, and reference lists of the remaining 65 references. Sixty-two records were excluded (Fig. 1). Finally, three RCTs, published between 1976 and 2002, were included in our meta-analysis: Pichler [16], Quinio et al. [17] and Pneumatikos et al. [18].

Within this search, two different concepts of antibiotic prophylaxis in polytrauma patients were identified: selective decontamination and the use of penicillin and ampicillin. The corresponding studies were grouped and analyzed separately.

### Study characteristics

Three RCTs [16, 17, 18] with a total of 256 participants (mean age 37.4 years, 81.6% male) with polytrauma met the criteria for inclusion and were analyzed in our meta-analysis. All included RCTs used a parallel-group design. Two studies compared selective decontamination with placebo [17, 18], whereas one study compared the usage of ampicillin and penicillin to standard of care [16]. The characteristics of the included studies are presented in Table 1.

**Table 1:** Study characteristics

Study (Year)	Study design	No of patients	Mean age ± SD (years)	Gender (m/f)	Intervention	Control
Quinio et al. (1996)	prospective, randomized, double-blind, placebo-controlled study	*N* = 148 SDD: *N* = 76 Placebo: *N* = 72	SDD: 35.5 **±** 16 Placebo: 33 **±** 15	SDD: 66/10 Placebo: 62/10	Selective digestive decontamination (SDD): a suspension of colistin sulfate (polymixin E) (10 mg/ mL), gentamicin (8 mg/mL), and amphotericinB (50 mg/mL) instilled through the nasogastric tube (10 mL) and nares (2 mL in each) 4x/day. An oral gel made of carboxymethylcellulose containing 2% of the same antibiotics was also applied 4x/day with a glove in the oropharyngeal cavity; about 15 mL of gel was used for one application.	Placebo
Pichler (1976)	Randomized, clinical study	*N* = 132 Antibiotics: *N* = 69 Standard care: *N* = 63	42.75	34/13	Antibiotic prophylaxis with 2 × 10 MegaE Natrium Penicillin G and 2 × 2 g Ampicilin	Standard care
Pneumatikos et al. (2002)	A pro-spective randomized, controlled, clinical study	*N* = 61 SDSA: *N* = 31 Placebo: *N* = 30	SDSA: 39.1 **±** 19.42 Placebo: 36.86 **±** 19	SDSA: 24/77 Placebo: 23/76	Selective decontamination locally in the subglottic area (SDSA): continuous infusion of a suspension containing 73 mg polymyxin E, 73 mg tobramycin, and 500 mg amphotericin B in 500 ml 0.9 saline solution at an infusion rate of 2 ml/h in the subglottic area for the entire period of the study	Placebo

NR = Not reported

### Risk of bias in studies

The overall risk of bias among the three RCTs was high for all outcomes due to a lack of a pre-specified analysis plan, no - or no sufficient - information regarding an appropriate analysis method, concealment, or blinding.

Assessment of reporting bias was not possible as there were not enough studies included.

### Results of individual studies

Antibiotic prophylaxis compared to placebo or standard care in adult polytrauma patients (Summary of findings Tables 2 and 3).

**Table 2:** Summary of findings table comparing of Ampicillin & Penicillin vs. standard care

Outcomes	No of participants (studies)	Certainty of the evidence (GRADE)	Relative effect (95% CI)	Anticipated absolute effects	
				Risk with standard of care	Risk difference with Ampicillin & penicilin
All-cause mortality	47 (1 RCT)	⨁◯◯◯ Very low^a, b^	**RR 1.04** (0.57 to 1.92)	458 per 1.000	**18 more per 1.000** (197 fewer to 422 more)

**Table 3:** Summary of findings table comparing of selective decontamination vs. placebo

**Outcomes**	**No of participants (studies)**	**Certainty of the evidence (GRADE)**	**Relative effect (95% CI)**	**Anticipated absolute effects**	
				**Risk with placebo**	**Risk difference with selective decontamination**
All-cause mortality	209 (2 RCTs)	⨁◯◯◯ Very low^a, b,c^	**RR 1.01** (0.55 to 1.85)	167 per 1.000	**2 more per 1.000** (75 fewer to 142 more)
Duration of mechanical ventilation	209 (2 RCTs)	⨁◯◯◯ Very low^a, c^	-	The mean duration of mechanical ventilation was **8.05** days	MD **1.22 days higher** (1.41 lower to 384 higher)
ICU length of stay (ICU LoS)	148 (1 RCT)	⨁◯◯◯ Very low^a, d^	-	The mean ICU length of stay was **15.7** days	MD **0.3 days higher** (3.99 lower to 4.59 higher)
Ventilator associated pneumonia (VAP)	61 (1 RCT)	⨁◯◯◯ Very low^a, d^	**RR 0.30** (0.13 to 0.72)	533 per 1.000	**373 fewer per 1.000** (464 fewer to 149 fewer)

CI: confidence interval; MD: mean difference; RR: risk ratio

a. High risk of bias due to no pre-specified analysis plan, no information regarding concealment or blinding and no information regarding analysis method

b. Inconsistency due to inconsistent direction

c. Imprecision due to few patients

d. Imprecision due to few patients and only one study

### All-cause mortality

#### Ampicillin & penicillin vs. standard care

**Fig. 2:**
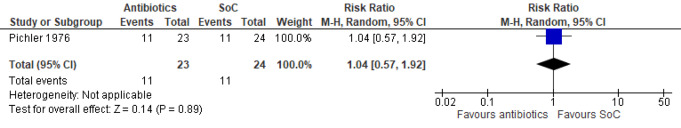
Forest plot describing the difference between antibiotics compared to standard care regarding all-cause mortality

One study reported all-cause mortality for 47 participants (Fig. 2). We found that penicillin and ampicillin contribute little or not to all-cause mortality compared to standard care (RR 1.04, 95% CI 0.57–1.92; risk difference (RD) 18 more per 1000, 95% CI 197 fewer to 422 more; 1 study, 47 participants; very low certainty of evidence). The reason for downgrading was imprecision due to only one study, small number of participants, and a high risk of bias.

#### Selective decontamination vs. placebo

**Fig. 3:**
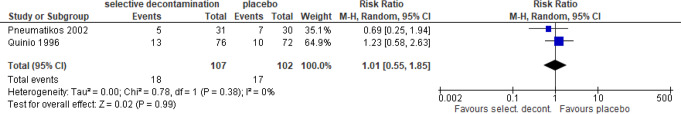
Forest plot describing the difference between selective decontamination compared to placebo regarding all-cause mortality

Two studies reported all-cause mortality for 209 participants (Fig. 3). We found that selective decontamination makes little or no difference to all-cause mortality compared to placebo (RR 1.01, 95% CI 0.55–1.85; risk difference (RD) 2 more per 1000, 95% CI 79 fewer to 150 more; 2 studies, 209 participants; *I*^2^ = 0%; very low certainty of evidence). The reasons for downgrading were a high risk of bias, indirectness due to inconsistent direction of point estimates, and imprecision due to the small number of participants.

### Clinical status: duration of mechanical ventilation

**Fig. 4:**
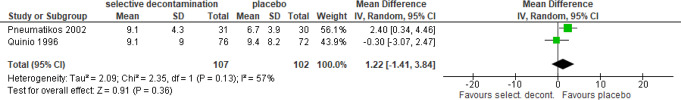
Forest plot describing the difference between selective decontamination compared to placebo regarding duration of mechanical ventilation

Two studies reported duration of mechanical ventilation for 209 participants (Fig. 4). We found that selective decontamination makes little or no difference to duration of mechanical ventilation compared to placebo (mean difference (MD) 1.22 days higher, 95% CI 1.41 lower to 384 higher; 2 studies, 209 participants; *I*^2^ = 57%; very low certainty of evidence). The reason for downgrading was imprecision due to the small number of participants and a high risk of bias.

### ICU length of stay

**Fig. 5:**
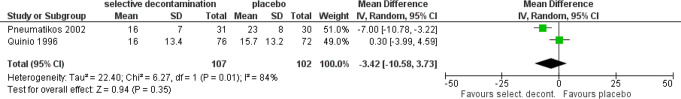
Forest plot describing the difference between selective decontamination compared to placebo regarding ICU length of stay

Two studies reported ICU length of stay for 209 participants (Fig. 5). We found that selective decontamination makes little or no difference to ICU length of stay compared to placebo (MD -3.43 days lower, 95% CI -10.58 lower to 3.73 higher; 2 studies, 209 participants; very low certainty of evidence). The reason for downgrading was imprecision due to the small number of participants and a high risk of bias.

### Ventilator associated pneumonia (VAP)

**Fig. 6:**
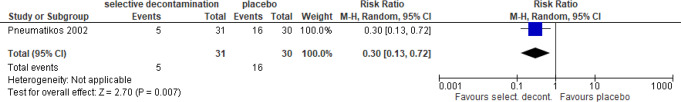
Forest plot describing the difference between selective decontamination compared to placebo regarding VAP

One study reported VAP for 61 participants (Fig. 6). We found that selective decontamination probably decreases the risk of VAP compared to placebo (RR 0.30, 95% CI 0.13–0.72; risk difference (RD) 373 fewer per 1000, 95% CI 464 fewer to 149 fewer; 1 study, 61 participants; very low certainty of evidence). The reason for downgrading was imprecision due to only one study, the small number of participants, and a high risk of bias.

We found no data regarding serious adverse events, adverse events, and quality of life.

## Discussion

In this review, three RCTs comparing antibiotic prophylaxis with placebo or standard care in 256 adult polytrauma patients were included. The results indicate no significant differences in all-cause mortality, duration of mechanical ventilation, and ICU length of stay when comparing antibiotic prophylaxis to placebo or standard care. However, selective decontamination was associated with a reduction in the risk of VAP, highlighting its potential role in specific patient populations.

These findings align with those of prior systematic reviews, including Minozzi et al. [20], who demonstrated that topical prophylaxis reduces respiratory infections in mechanically ventilated patients, although mortality benefits were only observed with a combination of topical and systemic antibiotics. D’Amico et al. [8] reported benefits of combined antibiotic prophylaxis in reducing respiratory infections, but also regarding overall mortality, in critically ill patients. Nathens et al. [19] further emphasized that selective decontamination of the digestive tract reduces mortality in critically ill surgical patients, but not in medical patients, highlighting the variability of its effects across patient populations.

While the results of this review suggest that antibiotic prophylaxis has no significant effect on mortality or clinical outcomes in polytrauma patients, certain clinical scenarios merit consideration. In particular, antibiotic prophylaxis may be beneficial in high-risk polytrauma patients requiring prolonged mechanical ventilation or immunosuppressants. The potential role of selective decontamination in reducing the risk of VAP is noteworthy, especially in settings where the prevalence of infections is high. However, these applications must be carefully weighed against the risks of antibiotic resistance and adverse effects. Tailored approaches based on local epidemiological data and individual patient profiles may optimize the use of antibiotic prophylaxis.

Despite these considerations, this systematic review has several limitations. The scarcity of high-quality RCTs, with one study dating back nearly 50 years, raises concerns regarding the applicability of these findings to contemporary clinical settings. Furthermore, the very low certainty of evidence, due to a high risk of bias among the studies and the small number of participants, weakens the strength of the conclusions. These limitations significantly restrict the applicability of these findings to contemporary clinical settings.

Given these limitations, this systematic review highlights the urgent need for more robust, high-quality RCTs to provide more robust evidence. Future research should explore variations in antibiotic prophylaxis approaches, patient subgroups, and contemporary clinical practices to better inform decision making. While this review provides a comprehensive synthesis of the available evidence, it underscores the importance of cautious interpretation and the need for further studies to guide clinical practice. As the body of evidence evolves, clinicians must balance the potential benefits of antibiotic prophylaxis against its associated risks and individual patient needs to adapt guidelines based on the emerging evidence.

## Conclusion

The results indicate that antibiotic prophylaxis has no significant effect on mortality and clinical status compared with placebo or standard care in adult polytrauma patients but may reduce the risk of VAP. However, the evidence is outdated and of very low certainty, with insufficient data to draw definitive conclusions regarding efficacy. Therefore, high-quality, up-to-date research is urgently needed to support clinical decision-making, and current interpretations should be treated with caution.

## Data Availability

Additional study data are available from the corresponding author.

## Appendix no 1. Search strategy

**Search section: Evidence Syntheses**.

**1. PubMed**.

#1 “Multiple Trauma“[mh] OR “Trauma Centers“[mh] OR polytrauma*[tw] OR “multiple trauma*“[tw] OR “major trauma*“[tw] OR “multiple injur*“[tw] OR “multisystem trauma“[tw] OR “multi-system trauma“[tw] OR “multisystem injur*“[tw] OR “multi-system injur*“[tw] OR “multitrauma“[tw] OR “multi-trauma“[tw] OR “life-threatening trauma*” [tw] OR “trauma patient*“[tw] OR “trauma population*“[tw] OR “trauma care“[tw] OR “trauma team*“[tw] OR “trauma cent*“[tw] OR “trauma unit*“[tw] OR “trauma resuscitation unit*“[tw] OR “Critical Illness“[mh] OR “Critical Care“[mh: noexp] OR “critical illness*“[tw] OR “critical care“[tw] OR “intensive care“[tw] OR “critically ill“[tw] OR “acutely ill“[tw] OR (blunt[tiab] AND trauma[tiab])

(379’990)

#2.

“Antibiotic Prophylaxis“[mh] OR ((“Anti-Bacterial Agents“[mh]) AND (“Wound Infection/prevention and control“[mh] OR “Sepsis/prevention and control“[mh])) OR “antibiotic prophylaxis“[Title/Abstract:~3] OR “antibiotics prophylaxis“[Title/Abstract:~3] OR “antibiotics prophylactic“[Title/Abstract:~3] OR “antibiotic prophylactic“[Title/Abstract:~3] OR “antibacterial prophylaxis“[Title/Abstract:~3] OR “anti-bacterial prophylaxis“[Title/Abstract:~3] OR “prophylactic antibacterial“[Title/Abstract:~3] OR “prophylactic anti-bacterial“[Title/Abstract:~3] OR “antibiotic premedication“[Title/Abstract:~3] OR “antibiotic premedications“[Title/Abstract:~3] OR “antibiotics premedication“[Title/Abstract:~3] OR “antibacterial premedication“[Title/Abstract:~3] OR “antibacterial premedications“[Title/Abstract:~3] OR “anti-bacterial premedication“[Title/Abstract:~3] OR “anti-bacterial premedications“[Title/Abstract:~3] OR “sepsis prevention“[Title/Abstract:~3] OR “antibiotic stewardship“[tw].

(35’716)

#3.

systematic[sb].

#4.

#1 AND #2 AND #3.

(78)

**Search section: RCTs**.

#5.

*[Cochrane RCT-filter*,* sensitivity max. version]* [21] (randomized controlled trial[pt] OR controlled clinical trial[pt] OR randomized[tiab] OR placebo[tiab] OR drug therapy[sh] OR randomly[tiab] OR trial[tiab] OR groups[tiab]) NOT (animals[mh] NOT humans[mh])

#1 AND #2 AND #5.

(953)

### 2. Cochrane Central Register of controlled trials via Cochrane Library

#1.

(polytrauma OR “multiple trauma*” OR “major trauma*” OR “multiple injur*” OR “multisystem trauma” OR “multi-system trauma” OR “multisystem injur*” OR “multi-system injur*” OR multitrauma OR multi-trauma OR “life-threatening trauma*” OR “trauma patient*” OR “trauma population*” OR “trauma care” OR “trauma team*” OR “trauma cent*” OR “trauma unit*” OR “trauma resuscitation unit*” OR “critical illness*” OR “critical care” OR “intensive care” OR “critically ill” OR “acutely ill”):TI, AB, KY.

#2.

((antibiotic* OR antibacterial OR anti-bacterial) NEAR/3 (prophylaxis OR prophylactic OR premedication*)):TI, AB, KY.

#3.

#1 AND #2 (206).

## Abbreviations

CI: Confidence interval
ICU: Intensive care unit
MD: Mean difference
MH: Mantel–Haenszel
MOF: Multiple organ failure
RCT: Randomized controlled trial
RD: Risk difference
RoB: Risk of bias
RR: Risk ratio
SDD: Selective decontamination of the digestive tract
SDSA: Selective decontamination locally in the subglottic area
SIRS: Systemic inflammatory response syndromes
SoC: Standard of care
VAP: Ventilator associated pneumonia
